# Reconstruction of the pollution history of alkylphenols (4-tert-octylphenol, 4-nonylphenol) in the Baltic Sea

**DOI:** 10.1007/s11356-016-6262-8

**Published:** 2016-03-02

**Authors:** Bożena Graca, Marta Staniszewska, Danuta Zakrzewska, Tamara Zalewska

**Affiliations:** Department of Marine Chemistry and Environmental Protection, Institute of Oceanography, University of Gdansk, Al. Marszałka Piłsudskiego 46, 81-378 Gdynia, Poland; Institute of Meteorology and Water Management, National Research Institute, Maritime Branch, Waszyngtona Str. 42, 81-342 Gdynia, Poland

**Keywords:** Alkylphenols, 4-nonylphenol, 4-tert-octylphenol, Sediment cores, Pollution reconstruction, Baltic

## Abstract

This paper reports the reconstruction of the pollution history of 4-tert-octylphenol (OP) and 4-nonylphenol (NP) in the Baltic Sea. Alkylphenols are endocrine-disrupting compound and therefore toxic to aquatic organisms. Sediment cores were collected from regions with relatively stable sedimentation conditions. The cores were dated by the ^210^Pb method. The OP and NP were determined using HPLC-FL. The highest inventory of these compounds was observed in the Gotland Deep (610 μg m^2^ of NP and 47 μg m^2^ of OP) and the lowest—on the slope of the Gdansk Deep (24 μg m^2^ of NP and 16 μg m^2^ of OP). Such spatial distribution was probably, among other factors, the result of the uplift of the sea floor. The pollution trends of OP and NP in sediments coincided with the following: (1) the beginnings of eutrophication (1960s/1970s of the twentieth century) and (2) strong increase in the areal extent and volume of hypoxia and anoxia in the Baltic (present century).

**Electronic supplementary material:**

The online version of this article (doi:10.1007/s11356-016-6262-8) contains supplementary material, which is available to authorized users.

## Introduction

Since the 1980s of the last century, 4-tert-octylphenol (OP) and 4-nonylphenol (NP) have been of environmental concern because of their toxicity for aquatic organisms (McLeese et al. 1981; Comber et al. 1993), estrogenic properties (Soto et al. 1991; Jobling and Sumpter 1993; Jobling et al. 1996) and widespread contamination. NP and OP belong to the broader group of compounds known as alkylphenols. They have been used for decades (Fiege et al. 2000) and the list of their applications is very long (COHIBA 2011a, b; OSPAR 2009; HELCOM 2009; 2010). The NP is mainly used for the synthesis of alkylphenol ethoxylates (NPEs). This compound belongs to the world’s third largest group of surfactants with various industrial, institutional and household applications. The OP is also used in the synthesis of alkylphenol ethoxylates (OPEs). However, its main application is the production of phenolic resin, predominantly as a tackifier in the vulcanization process for the manufacture of rubber tires. Generally, the OP, NP, and their ethoxylates are used in the following: textile industry, production of plastics, water-based paints, herbicides, pharmaceuticals, cosmetics and washing agents, as well as in pulp and paper processing and steel manufacturing (COHIBA 2011a, b; Jahan et al. 2008). Such extensive use causes NP and OP as widespread contaminants in natural environment. The main sources of OP and NP and their parent compounds, alkylphenol polyethoxylates (Ahel et al. 1994a; 1994b; Ying et al. 2002) for the marine environment are municipal and industrial wastes. Some of the authors pointed the atmospheric transport of alkylphenols (David et al. 2009; Ebinghaus and Xie 2006; Lewandowska et al. 2012). Due to the hydrophobic nature of OP and NP, marine bottom sediments should be considered as a sink for these constituents. Anoxic regions of the sea bottom in particular are likely to accumulate alkylphenols because anoxic condition preserves OP and NP and favors formation of these constituents by decomposition of their ethoxylates (Ying et al. 2002).

In this study, we examined sediment age and concentration of alkylphenols in sediment cores from deep-water areas of the Baltic Sea. In these areas, relatively stable sedimentation occurs and sediment composition reflects long-term trends in natural and anthropogenic terrestrial runoff and atmospheric deposition. Dating of sediments combined with the measurements of chemical composition of sediment cores have been frequently used to reconstruction pollution history in industrialized countries (e.g., Kumata et al. 2000; Van Metre and Mahler 2005; Isobe et al. 2001). It is crucial for the understanding of pollution mechanisms and for establishing effective countermeasures against pollution.

The Baltic Sea is the largest brackish water body in the world. The factors such as restricted water exchange, water stratification, oxygen deficiency in deep waters, shallow depths (mean depth = 53 m), and a large ratio of catchment area to the surface of the sea (4:1), cause the Baltic Sea to be sensitive to human pressure (Håkanson and Bryhn 2008; Wulff et al. 2001). Agricultural and industrial catchment areas have led to considerable degradation of the sea. Accurate information about the consumption and production of NP, OP and their ethoxylates in the Baltic Sea catchment as well as the runoff of these components in to the sea is lacking (HELCOM 2010). Since 2005, the NP and nonylphenol ethoxylates are restricted from use in cleaning products at concentrations exceeding 0.1 %, by the EU Directive (HELCOM 2010; Cox and Drys 2003). Apart from this, some European countries have introduced additional bans and restrictions on the use of NP and OP. However, neither their production and import, nor the import of products containing these compounds is prohibited. Therefore, it is not surprising, that alkylphenols were detected in water, certain organisms, and sediments (Staniszewska et al. 2014; 2015; Koniecko et al. 2014) of the Baltic Sea. Until now, NP and OP were determined both in coastal and in offshore regions of the Baltic Sea but only in the surface layer of sediments (1 or 2 cm). It has given just a “snapshot” of the current pollution status and no information on the historical trend of pollution in the region has been available.

## Materials and methods

### Sample collection and cores processing

**Fig. 1:**
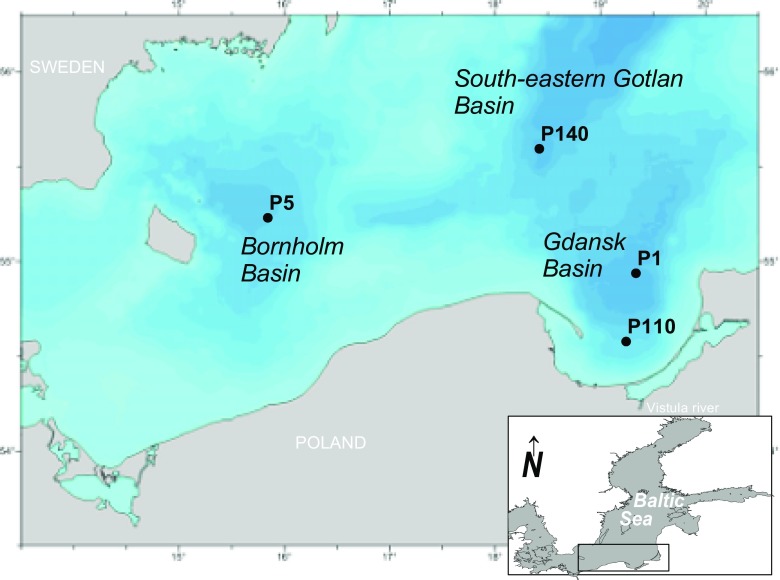
Map of the study area

Sediment samples were collected at four sampling stations located in the southern Baltic Sea: on the S-W slope of the Gotland Deep (P140, depth = 88 m); in the Bornholm Deep (P5, depth = 86 m), in the Gdansk Deep (P1, depth = 106 m); and on the slope of the Gdansk Deep (P110, depth = 70 m) (Fig. 1, Table 1). The samples were taken onboard r/v Baltica, with a Niemisto-type corer, in June 2014.

**Table 1:** Characteristics of sediments and near-bottom waters in the area of study

Region	Station	Water depth (m)	Salinity in near-bottom water	Oxygen (ml l^−1^)	Sediment type*
Gdansk Deep	P1	106	12.26	2.03	Clays and silty clays
Slope of the Gdansk Deep	P110	70	9.72	3.97	Clays and silty clays
S-W Gotland Deep	P140	88	12.35	3.93	Silty clays
Bornholm Deep	P5	86	16.52	2.61	Clays and silty clays

*Kramarska et al., 1995

Six sediment cores were collected from each sampling area. Cores were divided into 1- or 2-cm layers within the uppermost 25 cm. The segments of equal depth from three cores were pooled together and frozen. Such procedure allows to get a weight of samples required to obtain assumed limit of quantification and the uncertainty of sediment dating method with ^210^Pb (Suplińska and Pietrzak-Flis 2008; Zalewska et al. 2015). Furthermore, vertical heterogeneity of the sediment chemical composition is included. Alkylphenol content and sediment age determination were done in the first set of three integrated cores. Granulometry, water content, and loss on ignition have been analyzed in the second set of three integrated cores from a given area. Additionally, the sediment core for Eh measurements was collected and cut into 2.5-cm layers (in the 0–10 cm section of the core) and into 5-cm layers (in the >10 cm section of the core).

### Sediment parameters: redox potential, granulometry, water content, loss on ignition

Laboratory analyses involved general sediment properties, such as water content (*W*), loss on ignition (LOI), redox potential (Eh), and grain-size distribution. Redox potential was measured as soon as possible after core retrieval, onboard the research vessel, using an Eh-meter (Microscale Measurements, The Netherlands, model: MB II mV). Slicing of sediment core lasted about 5 min. Each segments of sediment were placed in containers. Before closing, the containers were flushed with nitrogen. During the measurements, the needle of the electrode was inserted in the middle part of segment. Measurement of Eh in one segment lasted about 10–15 min (the stabilization time of the electrode).

Water content (*W* %) and loss on ignition (LOI %) were determined by drying the sediment samples to constant weight at 105 and 450 °C, respectively. Granulometric analysis was conducted by sieving dry sediment through a FRITSCH GmbH metal sieves set consisting of 2.00, 1.00, 0.50, 0.25, 0.125, and 0.063 mm net dimensions.

## Sediment age determination (sediment dating with ^210^Pb)

### Spectrometric analysis

The freeze-dried sediment samples were homogenized (slices from three integrated cores) prior to the exact analysis. The samples were placed in plastic containers.

The activity concentrations of ^210^Pb, ^137^Cs, ^214^Bi, and ^214^Pb were analyzed by high-resolution gamma spectrometry using an HPGe detector with a relative efficiency of 40 % and a resolution of 1.8 keV for the peak of 1332 keV of ^60^Co. The detector was coupled to an 8192-channel computer analyzer (GENIE 2000). The time of measurements was 80,000 s for each sample. ^210^Pb was determined by gamma emission at 46.5 keV, ^226^Ra was determined by the emission of its daughter nuclides ^214^Pb and ^214^Bi at 352 and 609 keV, respectively, and ^137^Cs was measured via its emission at 661.6 keV. A mixture of gamma-emitting isotopes-“mix gamma” (Isotope Production and Distribution Center, Swierk, Poland, BW/Z-62/27/07), was used for the calibration.

### Mass accumulation rate, linear accumulation rate, and sediment age calculations

Total amount of radioactive ^210^Pb isotope (^210^Pb_tot_) that can be found in sediments is the sum of ^210^Pb originating from the radioactive decay of radium (^226^Ra), which is called supported ^210^Pb (^210^Pb_supp_), and ^210^Pb originating from the atmospheric deposition, which is called unsupported or excess ^210^Pb_ex_. The activity of ^210^Pb_supp_ along the sediment profile is approximately constant, while the activity of ^210^Pb_ex_ decreases down the profile and this decrease is usually exponential. As a result, ^210^Pb_ex_ distribution along the sediment profile is used to calculate mass accumulation rate (MAR), linear accumulation rate (LAR), and the age of sediment. These calculations are based on constant flux/constant sedimentation rate (CF/CS) model) (Appleby 1997; Robbins 1978; Bierman et al. 1998; Szmytkiewicz and Zalewska 2014). Results of sediment dating were verified based on ^137^Cs profiles in sediment. The LAR expresses an average thickness of a layer that is formed within 1 year, while the MAR corresponds to an average mass of sediment deposited on the area of 1 m^2^ within 1 year. Concentrations of radioactive ^210^Pb_ex_ in the analyzed sediment layers were determined as the difference between concentrations of ^210^Pb_tot_ and ^214^Bi. The average concentration of ^214^Bi for all the analyzed layers in a core was assumed as the ^214^Bi value for a particular core.

### Alkylphenols

In the analysis of alkylphenols, the following reagents were used: water, acetonitrile, methanol HPLC grade (Merck), and high grade standards of 4-nonylphenol and 4-tert-octylphenol (>97 % of purity; Sigma-Aldrich). Lyophilized sediment samples (2 g from each slice of three integrated cores) were extracted twice with a mixture of deionized water and methanol (30:70) in an ultrasonic bath. The combined extracts were purified on SPE C18 columns using a method developed by Nunez et al. (2007) and Koniecko et al. (2014). Elution was carried out with methanol and acetonitrile. The obtained solutions were dried in a rotary evaporator and reconstituted in 200 μdm^3^ of acetonitrile. The final stage of the analysis of alkylphenols was conducted using a high-performance liquid chromatograph Dionex UltiMate 3000 with a fluorescence detector (exCitation *λ* = 275 nm, emission *λ* = 300 nm) and a Thermo Scientific HYPERSIL GOLD C18 PAH chromatography column (250 × 4.6 mm; 5 μm) in the mobile phase program (water/acetonitrile) in gradient conditions.

The linear correlation coefficient *r* for the analytical curves of working solutions (10, 25, 50, 75, and 100 ng cm^−3^) was 0.999. The limit of quantification (LOQ) was 0.08 ng g^−1^ dw for both alkylphenols. The background value was below the quantification level. Recovery of both studied compounds was determine in a sediment sample spiked with a known amount of the standard and mean value was 94 % (OP) and 81 % (NP). The precision, expressed as the relative standard deviation coefficient (RSD) for five repetitions of the same sample, was below 8 % for each of the compounds.

### NP and OP inventory of sediment

Based on the obtained results, inventories of NP and OP of the sediment were assessed. The inventory was defined as the total amount (μg) of pollutants per area (m^2^), deposited since the 1920s of the twentieth century. The inventories (*I*) were calculated according to Eq. 1:

1 $$ \mathsf{I}={\displaystyle {\sum}_{\mathsf{n}=\mathsf{1}}^{\mathsf{n}=\mathsf{1}\mathsf{0}\mathsf{or}\mathsf{12}\mathsf{or}\mathsf{13}}\left(\mathsf{100}\hbox{-} \mathsf{W}\right)\ast \mathsf{1}\mathsf{0}{\mathsf{0}}^{\hbox{-} \mathsf{1}}\ast \mathsf{h}\ast \mathsf{d}\ast \mathsf{C}} $$

where *I* = NP or OP inventory (μg m^2^), *n* = the number of sediment segments (depends on the sediment age), *W* = sediment water content (%), *h* = thickness of sediment layer (m), *d* = average sediment bulk density (260 10^5^ g m^−3^), and *C* = NP or OP concentration in given segment of sediment (ng g^−1^ dw).

### Statistical analysis

The statistical analysis and visualization of the results was carried out using the STATISTICA 10 by Stat Soft and Microsoft Office Excel 2007.

## Results

### Sediment characteristics

#### Water content

**Fig. 2:**
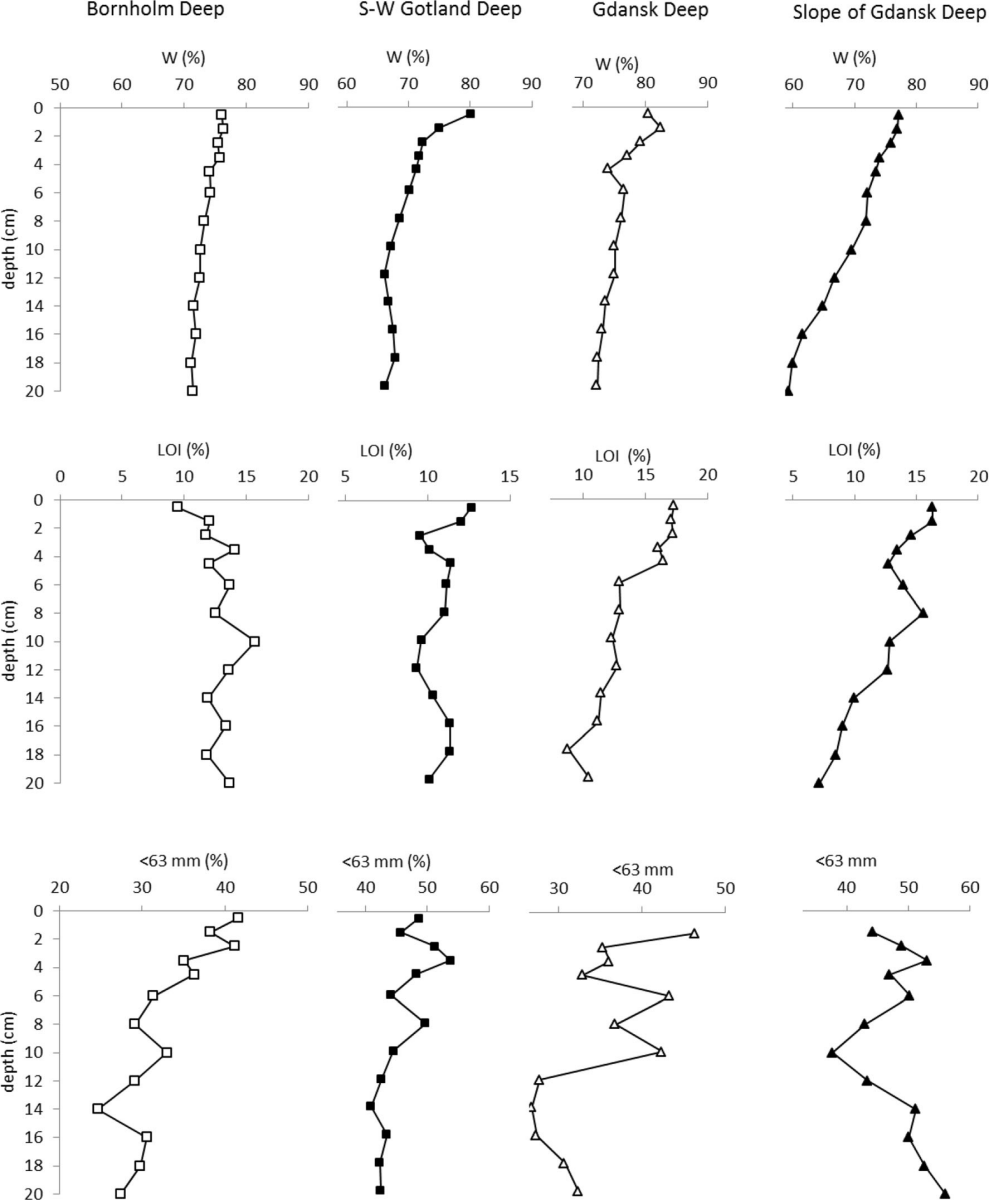
Profiles of water content (*W*), loss on ignition (LOI), and the percentage of <63 μm fraction in sediments

Water content of the sediments varied within a relatively narrow range, from 59 to 82 % (Fig. 2). The highest range of water content (from 55 to 79 %) was observed in the Gdansk Deep (station P1), the lowest one (from 71 to 76 %) in Bornholm Deep (station P5). In sediments of all studied stations, water content regularly decreased with sediment depth (Fig. 2).

#### LOI

LOI in the uppermost 1 cm of the sediment varied from 9.5 to 17.5 % (Fig. 2), and was never lower than 7.1 % in deeper sediment layers. A decrease in LOI down the sediment profile was typical in the Gdansk Deep (P1) and on its slope (P110). In the other regions, the changes of LOI in sediment profiles were irregular, without any general increasing or decreasing trends. In the area of the Gdansk Deep (P1) and its slope (P110), LOI values in surface sediments (0–3, 0–4 cm) were several percent higher than in the other areas (Fig. 2).

#### Contribution of the <0.063 mm fraction

Contribution of the <0.063 mm fraction varied from 25 to 57 %. In the first centimeter of the sediment, the fraction below 0.063 mm constituted from 42 to 47 % of all fractions. In all regions, expect of slope of Gdansk Deep, contribution of <0.063 mm fraction decreased irregularly with sediment depth (Fig. 2). At the slope of the Gdansk Deep (P110), the changes was irregularly with no clear trends. The highest range of <0.063 mm fraction (from 26 to 47 %) was observed in the Gdansk Deep (station P1).

#### Eh

**Fig. 3:**
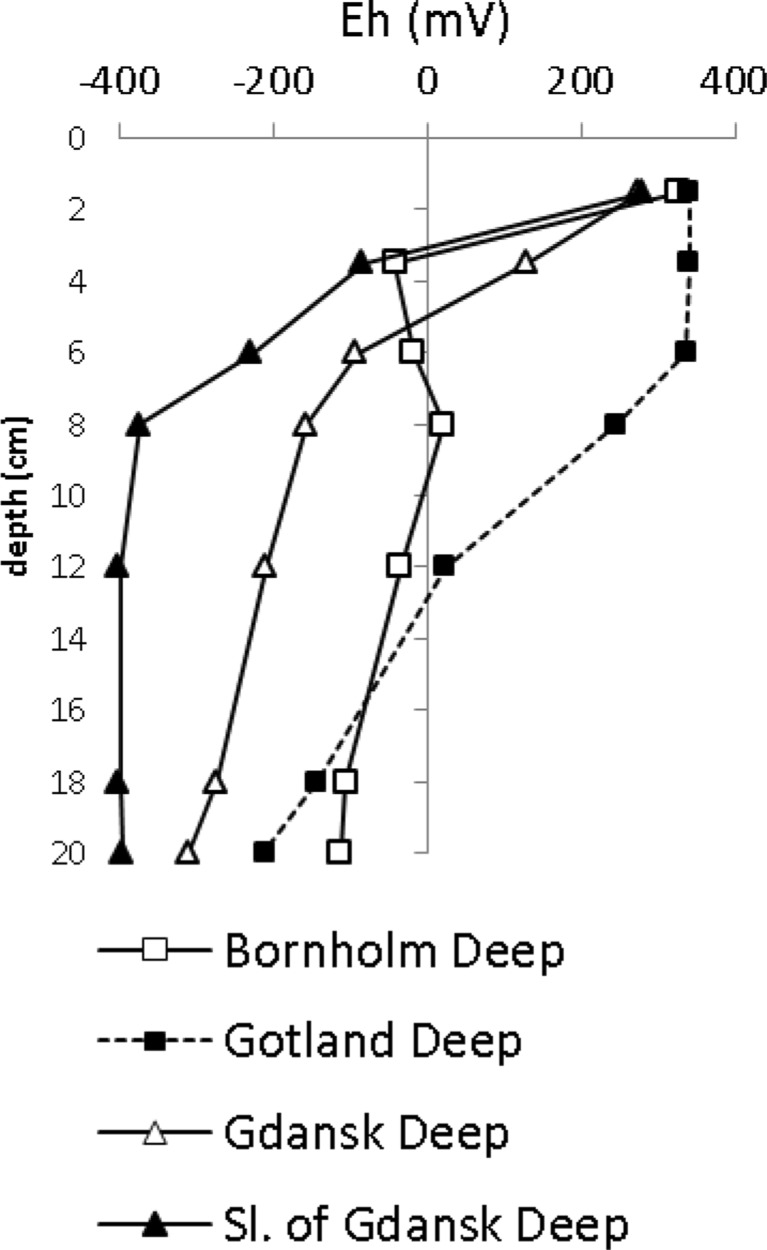
Profiles of Eh in sediments

The Eh potential of sediments decreased approximately exponentially in sediment profiles (Fig. 3). Reducing conditions (Eh <100 mV) were observed below the 3–4 cm of sediment, except for the station located on the slope of the Gotland Deep (P140), where they appeared below the layer of 7–9 cm below sediment surface (Fig. 3).

### Sedimentation rate

Linear and mass accumulation rates increased in the following order: Gdansk Deep, slope of the Gdansk Deep, S-W Gotland Deep, Bornholm Deep (Table 2). Profiles of ^210^Pb_exp_ and ^137^Cs, used for the calculation and verification of the obtained results, can be found in supplementary materials (Figs. 1S and 2S).

**Table 2:** Linear accumulation rate (LAR) and mass accumulation rate (MAR) in sediments of the study area

Region	Linear accumulation rate	Mass accumulation rate
	mm year^−1^	g m^−2^ year^−1^
Bornholm Deep	3.0	979
S-W Gotland Deep	2.0	800
Gdansk Deep	1.6	470
Slope of the Gdansk Deep	1.8	749

### Alkylphenols

#### NP concentrations

**Fig. 4:**
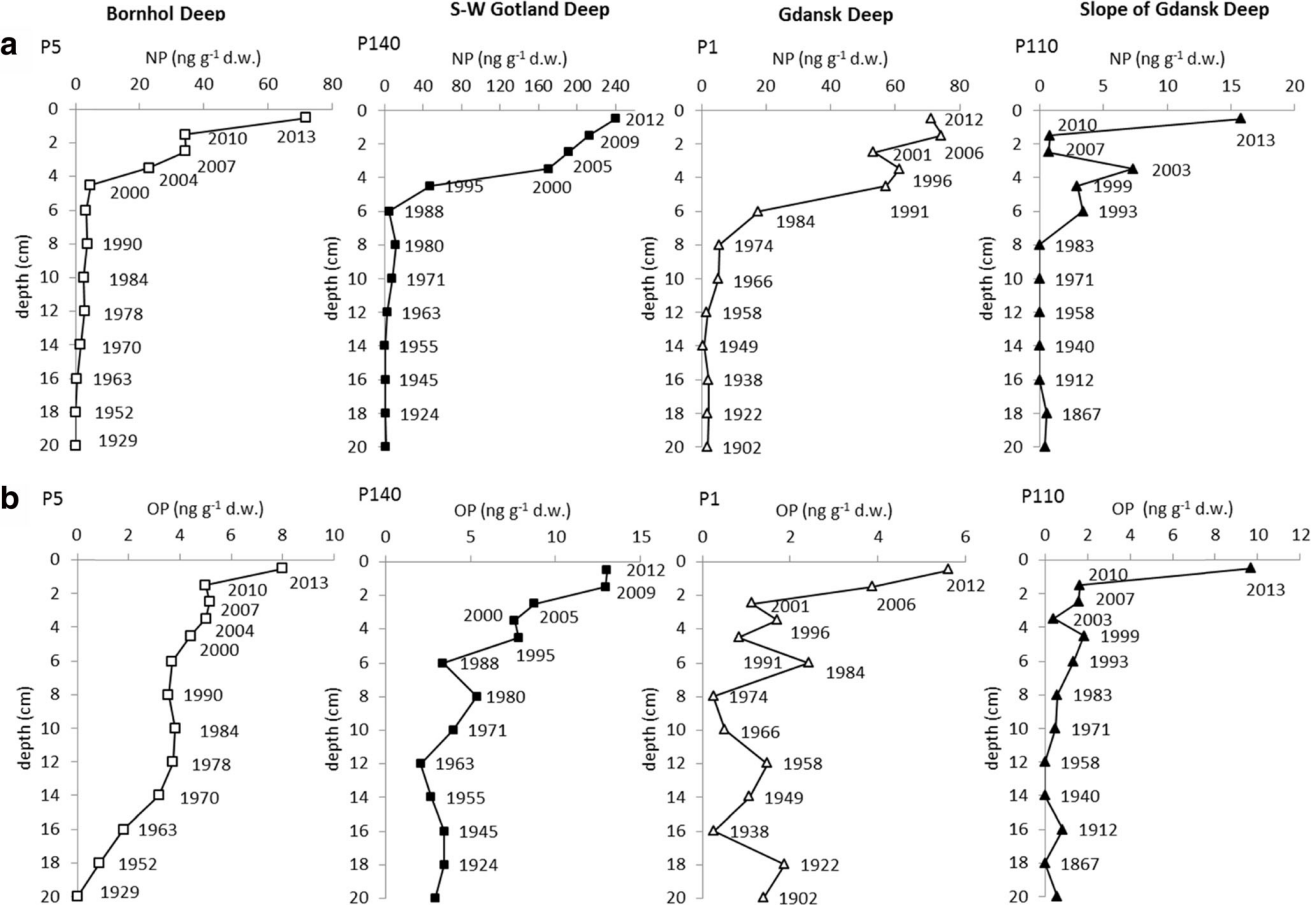
Profiles of **a** NP and **b** OP in sediments of the study area

NP concentrations ranged from <LOQ to 239.88 ng g^−1^ dw of sediment (Table 3). In the surface layer (0–1 cm), they varied between 15.80 and 239.88 ng g^−1^ dw (99.72 ± 97.07 ng g^−1^ dw). Clear spatial differences in the content of NP were observed in the layers from the sediment surface to the 5–7 cm below sea floor. The highest values were reported in sediments of the S-W Gotland Deep (P140) (Fig. 4a) and the lowest on the slope of the Gdansk Deep (P110). In sediments of the Gdansk Deep and Bornholm Deep, NP concentrations in surface sediments were similar, while in deeper sediment layers, higher values were measured in the area of the Bornholm Deep.

**Table 3:** The outcome of the statistical analysis of OP and NP content in sediments of the study area (sediment layer 0–25 cm)

	Region	Number	Mean ± SD	Min–max	Median	Lower quartile	Upper quartile
NP (ng g^−1^ dw)	Bornholm Deep	13	14.04 ± 21.50	ND–71.92	3.21	1.64	22.88
	S-W Gotland Deep	13	68.67 ± 95.50	0.43–239.88	8.09	1.51	170.08
	Gdansk Deep	13	27.39 ± 30.60	0.71–74.59	5.60	2.07	57.36
	Slope of the Gdansk Deep	13	2.47 ± 4.53	ND–15.80	0.59	0	2.96
OP (ng g^−1^ dw)	Bornholm Deep	13	3.71 ± 2.04	ND–7.99	3.72	3.18	5.00
	S-W Gotland Deep	13	5.96 ± 3.77	2.08–13.01	3.99	3.38	7.83
	Gdansk Deep	13	1.72 ± 1.52	0.25–5.61	1.39	0.83	1.88
	Slope of the Gdansk Deep	13	1.46 ± 2.55	ND–9.68	0.58	0.40	1.58

In the deepest sediment layers which, depending on the region, corresponded to the period from 1867 to 1929, NP concentrations ranged from <LOQ to about 2 ng g^−1^ dw. In the area of the Bornholm Deep and S-W Gotland Deep, NP concentrations increased up the sediment column, beginning from the depth corresponding to the early 1960s of the last century. A rapid increase in the concentrations was observed from the years 2000 and 1988, respectively, for the Bornholm Deep and for the S-W Gotland Deep. In the area of the Gdansk Deep, the concentrations started to increase in the end of the 1950s of the last century. The particularly strong growth of NP in this region began in the mid-1970s. On the slope of the Gdansk Deep, the increase started in 1983. In the first decade of the twenty-first century, a short-term decrease in the concentrations was reported in this region. However, since 2010, the NP levels have gone up again.

#### OP concentrations

OP concentrations were from <LOQ to 13.01 ng g^−1^ dw of sediment (Table 3). In the surface layer (0–1 cm) they ranged from 5.61 to 13.01 ng g^−1^ dw (9.07 ± 3.11 ng g^−1^ dw). Spatial differences in the content of OP were observed in the whole profile, and they were the greatest in top 5–7 cm (Fig. 4b). The highest values were determined in sediments of the S-W Gotland Deep. Relatively high values were also reported for sediments of the Bornholm Deep. In the area of the Gdansk Deep and the slope of the Gdansk Deep, OP concentrations were similar and, in most sediment layers, lower than in the other areas. The 0–1 cm sediment layer of the slope of the Gdansk Deep was the exception as the concentration of OP at that site amounted to 10 ng g^−1^ dw, which was lower only than the value measured in the surface layer of sediments in the S-W Gotland Deep.

In the deepest sediment layers which, depending on the region, corresponded to the period from 1867 to 1929, OP concentrations ranged from <LOQ in the area of the Bornholm Deep (P5) to about 3 ng g^−1^ dw in the area of the S-W Gotland Deep (P140). In sediments of the Bornholm Deep, Gotland Deep, and the Gdansk Deep (together with its slope), the concentration started to increase in the 1950s, 1960s, and 1970s of the last century, respectively. In all the areas, the strongest increasing tendency was observed for sediments of this century. Since 2000, the concentrations of OP have increased about two-, three-, six,- and fivefold in the sediments of the Bornholm Deep, S-W Gotland Deep, Gdansk Deep, and the slope of the Gdansk Deep, respectively.

There was no statistically significant dependences between the concentrations of alkylphenols and LOI (*r* = −0.12, *p* = 0.38 for OP and *r* = 0.00, *p* = 0.97 for NP). A relatively high positive linear correlation between redox potential and both alkylphenols, OP (*r* = 0.68, *p* < 0.001) and NP (*r* = 0.58, *p* = 0.001) was observed. Also, the relationship between the OP and NP was characterized by relatively high coefficient of linear correlation (*r* = 0.77, *p* < 0.001) (Fig. 2S).

## Discussion

### Regional variability, impact factors and inventories of alkylphenols

Due to the small amount of literature data, it is difficult to compare OP and NP levels obtained in this study with other open sea areas of the Baltic Sea. The NP concentrations obtained in the present study for surface layer of sediments are high as compared with concentration of NP in surface sediments from offshore sites of the Swedish sub-basins (Table 4). Moreover, the levels measured in the area of the Gotland Deep exceeded the predicted no-effect concentration (PNEC) value adopted by HELCOM (2010), which amounts to 180 ng g^−1^ dw. The PNEC is the concentration below which exposure to a substance is not expected to cause adverse effects. Presented value of PNEC (180 ng g^−1^ dw) is calculated as a product of a partition coefficient suspended matter-water and the PNEC in water, divided by the bulk density of suspended matter (for details see Anonymous 2005a; Lepper 2005). Usually, the PNEC is calculated using toxicity data. However, eco-toxicological data of PNEC for NP and OP, concerning the sediment and biota, are lacking. Calculation of PNEC based on the equilibrium partitioning method, has some limitations compared to PNEC estimate based on eco-toxicological data. The equilibrium partitioning approach only considers uptake via the water phase. However, uptake may also occur via other exposure pathways like ingestion of sediment and direct contact with sediment (Anonymous 2005b). Concentrations of NP obtained in the present study were also greater than, for example, the values in the surface layer of the German Bight sediments and lower than concentrations in most of the basins located along highly urbanized coasts of Asia and North America (Table 4).

**Table 4:** Concentration of 4-nonylphenol and 4-*tert*-octylphenol (ng g^−1^ dw) in surface layer of sediments in different bodies of water in the world

	Region	NP (ng g^−1^ dw)	OP (ng g^−1^ dw)	References
Europe	Costal Baltic Sea, The Gulf of Gdańsk (Poland)	0.08–49	0.08–250	Koniecko et al. 2014
	Offshore Baltic Sea	<3 to 65 (not detected at 10 out of 14 investigated sits)	<3–110 (not detected at 5 out of 14 sites)	Cato and Kjellin 2005; 2008
	Offshore Baltic Sea (the Gotland, Bornholm, Gdansk Deeps)	16–240	5.6–13	This work
	Costal Baltic Sea Espoo (Finland)	2.2	<0.2	Hansen and Lassen 2008
	Costal Baltic Sea Oslo Fjord (Norway)	<0.2	<0.2	Hansen and Lassen 2008
	North Atlantic, Faroe Islands	0.7–3.1	24.8	Hansen and Lassen 2008
	Costal North Sea, German Bight (Germany)	<10–55		Bester et al. 2001
	Costal North Sea, Dutch estuaries (The Netherland)	0.9–1080		Jonkers et al. 2003
	Costal Aegean Sea, Thermaikos Bay (Greece)	266		Arditsoglou and Voutsa 2012
	Costal Adriatic Sea, Venice Lagoon (Italy)	47–132		Pojana et al. 2007
	Mediterranean Sea (Spain)	18–590		Petrovic et al. 2002
	Coast Atlantic (Spain)	23–1050		Petrovic et al. 2002
	Coast Atlantic, Cadiz Bay (Spain)	13–225		Lara-Martin et al. 2006
Asia	Coast China Sea (Taiwan)	130–190	27–49	Chen et al. 2005
	Coast China Sea, Masan Bay (Korea)	113–3890	4–179	Khim et al. 1999
	Coast Pacific Ocean, Tokyo Bay (Japan)	120–640	6–100	Isobe et al. 2001
	Coast Pacific Ocean, Tokyo Bay (Japan)	142–20,700		Kurihara et al. 2007
North America	Coast Caribbean Sea, Jamaica Bay (USA)	7–13,700	2.4–45	Ferguson et al. 2001
	Coast Pacific Ocean, Southern California Bight (USA)	130–3200	1.9–8.2	Schlenk et al. 2005
	Coast Atlantic, Marsh–estuaries Savannah (USA)	10.0–18	2.6–6.9	Senthil Kumar et al. 2008

The concentrations of OP obtained in the present study (5.61 to 13.01 ng g^−1^ dw) for surface sediments (0–1 cm) were average or high as compared with the values in the surface layer of offshore Baltic sediments (Table 4). In all areas investigated in this study, OP concentrations exceeded the predicted no-effect concentration (PNEC) of 3.4 ng g^−1^ dw adopted by HELCOM (2010), estimated on the basis of the equilibrium partitioning method (Anonymous 2005b). However, the concentrations were much lower than, for example, in sediments off the coast of Japan and America, and comparable to those recorded in the Pearl River Estuary and South China Sea (Table 4).

The studies on the kinetics of OP sorption in sediments showed that it is a two-step process. The first step includes fast sorption on the surface. After that, a slow interparticle sorption takes place (Zhou 2006). Probably, alkylphenol fraction which is interpartically sorbed cannot be entirely measured using a standard extraction procedure. This was indicated in the study by Milinovič et al. (2015), who developed the NP sequence analysis. Their research showed that in sediments of the Bohai Bay (North China Sea), non-extractable (interpartically sorbed) NP constituted 38–99 % of total NP. In the present work, similarly as in other previous studies, alkylphenols were extracted using the standard solvent mixture (methanol and water). Hence, it is probable that the results obtained so far (in this study and in similar previous studies) were underestimated as they did not include interpartically sorbed fraction. It is therefore important to apply new sequence analysis in future studies.

Numerous studies, especially experimental ones, indicated that the fraction of organic carbon is an important parameter that can have an influence on the sorption of alkylphenols and other organic contaminants in sediments (Milinovič et al. 2015; Mayer 1993; John et al. 2000). Lack of significant relationships between LOI and alkylphenols in presented study was presumably the result of the complex nature of processes controlling alkylphenol sorption in environmental condition. It can be assumed that in the case of sediments similar to those in the study area, fine-grained and rich in organic matter, the composition of organic matter, e.g., black carbon content (Koniecko et al. 2014), occurrence of colloids (Zhou 2006), and degree of organic matter decomposition, are crucial for the sequestration of NP and OP in sediments. The effect of the latter seems to be confirmed by a relatively high positive linear correlation between redox potential and both alkylphenols. But this is probable connected to the fact that the amount of OP and NP is very low in the deep sediment layers, since they were sedimented before the onset of alkylphenols industrial production and the Eh is always negative because are anoxic sediment layer. When Eh increases toward sediment surface because of a higher oxygen availability, NP and OP does it because their sedimentation rate increase since are more recent layers.

Relatively high relationship between OP and NP observed in this study may indicate the same or similar sources of these compounds to the marine environment. However, based on the scatter plot, we can see that the high value of *r* coefficient was caused by only four results (Fig. 3S). The NP is supplied to the marine environment probably mainly with sewage containing NPEs. In the case of OP, its important source can be dust formed by abrasion of tires. According to COHIBA (2011a), more than 50 % of the OP external load is introduced to the Baltic Sea in this way, although this is disputed by tire manufacturers. The producers claim that OP is used in resins inside the layers and not on the tire tread. However, when tires are mechanically crushed, OP can be potentially released. The set of results obtained in the present study did not allow any definite conclusions to be drawn on dependences between NP and OP, more results and further research is needed.

The concentration of organic contaminants in sediments depends, among others, on their supply from the catchment area, their sorption and degradation processes in sediments, and hydrodynamic conditions. The hydrophobic nature of the analyzed compounds (*K*_o/w_ = 4.12 and 4.48, for OP and NP, respectively) causes that they are probably supplied to the bottom mainly with the suspended matter. In the study area, its inflow to the sediments occurs through the vertical as well as horizontal transport. Contaminants which reach bottom sediments are “diluted” by bioturbation and total sediment accumulation rates (Jonsson 2000). In order to compare the investigated sites in terms of the final sink for NP and OP, the inventories of OP and NP were calculated (Table 5). That approach enabled to disregard the influence of dilution and allowed a real comparison of the deposited amounts of alkylphenols.

**Table 5:** Calculated inventories (*I*) of NP and OP

Region	NP	OP
	μg m^2^	
Bornholm Deep	126	47
S-W Gotland Deep	610	74
Gdansk Deep	210	17
Slope of the Gdansk Deep	24	16

The S-W Gotland Deep showed clearly higher amounts of the measured contaminants than the other areas. In contrast, the inventory of NP was almost twice of that reported in the Bornholm Deep. On the contrary, the inventory of OP was three times lower than in the Bornholm Deep. The smallest amounts of OP and NP were deposited on the slope of the Gdansk Deep. The spatial variability of the pollutant inventories did not match the observed linear and mass accumulation rates. This indicates differences in the quality of material supplied to the bottom and/or differences in the retention of alkylphenols in the study area. The main reason for the observed variability could be the uplift of the Baltic Sea floor. The land uplift in the Baltic region varies from about 9 mm year^−1^ in the northern part of the sea to about 0 in the southern-central part of the Baltic Proper (Håkanson and Gyllenhammar 2005). It was shown that land uplift may be responsible for the settling of 50–80 % of the materials below the wave base in the open Baltic Proper (Jonsson et al. 1990; Jonsson 1992; Blomqvist and Larsson 1994; Eckhéll et al. 2000). As a result, at the edge of the Gotland Basin, on the slope of the Gotland Deep (station P140), greater amounts of material from land uplift can be potentially accumulated than in the other study areas. It is worth noting that high concentrations of alkylphenols in the surface sediments of deep-water Gotland Basin areas were observed earlier by Cato and Kjellin 2005. The Gotland Basin has the largest catchment area among all the regions of the Baltic Sea and receives water from highly industrialized Scandinavian countries where, for example, paper and pulp have been produced for decades. This region is also characterized by high intensity of sea transport (off the Swedish coast) (HELCOM 2009). Oil spills and leaching of OP and NP from paints covering the hulls of ships could be a source of alkylphenols to the marine environment (COHIBA 2011a; 2011b). Moreover, extensive blooms of blue-green algae take place regularly in the Gotland Basin, and in its deep-water zones, we can observe permanent oxygen deficiency (e.g., Carstensen et al. 2014). It favors the accumulation of alkylphenols in sediments and formation of OP and NP by decomposition of their ethoxylates (Ying et al. 2002). For 4-nonylphenol, 401 days are required in anaerobic conditions for a complete breakdown of this compound (Heinis et al. 1999). When oxygen is involved, the decomposition of 4-nonylphenol occurs more rapidly, with nearly 50 % of this compound being decomposed within 10 days (Ying et al. 2003).

### Historical trends of alkylphenol contamination in sediments

The synthesis of first phenolic resins was developed in the years 1907–1911. However, octyl- and nonylphenol ethoxylates started to be produced on an industrial scale in the 1930s and 1940s, respectively. Since then, the production increased exponentially, especially that of nonylphenol (Fiege et al. 2000). The present study indicated that the contamination of sediments by the NP was pronounced by its increased concentrations in sediments of the accumulation bottom about 20 years after its mass production had started, namely in the 1960s. The 1960s of the twentieth century was a period when first symptoms of eutrophication in the form of significant long-term decreasing trends in oxygen concentrations in deep waters (Fonselius 1969) and increasing concentrations of phosphates appeared in the Baltic Sea. It can be assumed that the increase in the supply of organic matter to the sediments and oxygen depletion resulting from eutrophication, indirectly enhanced alkylphenol accumulation in sediments (Ying et al. 2002). The slope of the Gdansk Deep was the only area where NP accumulation began much later (in the 1980s of the twentieth century). This area is the shallowest of all the investigated in the present study. Due to its depth (about 70 m), the bottom of the slope of the Gdansk Deep can be influenced by wind mixing, and thus this area is less conducive to accumulation and long-term oxygen deficiency. Lower accumulation of the analyzed compounds in the sediments of the Gdansk Deep slope could also be the result of significantly lower than in the other areas salinity of near-bottom waters. Experimental studies showed that the sorption of OP is enhanced in the presence of salts (Zhou 2006).

In many works, it was emphasized that NP and OP are of anthropogenic origin (e.g., Chen et al. 2005). Therefore, their presence in sediment layers deposited before the period of their production is surprising. In the present study, their concentrations reached relatively high levels in those layers, particularly in the case of OP. Both NP and OP were detected in European groundwaters (Loos et al. 2010), which indicates their ability to infiltrate despite very high, especially for NP and *K*_o/c_ partition coefficient. It is possible that the mobility of alkylphenols in fine-grained sediments is stimulated by reduction conditions and acidic conditions, similarly as in the case of metals or phosphates (Chuan et al. 1996). Theoretically, OP and NP presence in sediment layers deposited before the period of their production could be also the result of bioturbation or bioirrigation. But in the deep water of the Baltic Sea, macrobenthic organisms are limited and even lacking (Warzocha 1995).’

According to UBA (2006), the production volumes of NP in the EU were reduced by 50 % since 2005. The NP sediment profiles obtained in the present study did not indicate any response of the ecosystem to decreased external loading of that compound, which suggests potentially large internal load of NPs in the Baltic Sea. The limited exchange of water, as in the study area, is generally conducive to long retention of pollutants. In the present century, an increase in the accumulation was also observed for OP, which production, according to UBA (2006), remained at a similar level to 2005. Increased accumulation of OP and NP in last decade in deep-water areas should be associated with drastic changes in oxygen conditions. Based on the oxygen data from the period of 1960–2011, it was observed that a distinct regime shift occurred in 1999. Before that shift, from 1960 to 1999, hypoxia affected large areas and volumes while anoxic conditions affected only minor deep areas of Baltic Sea. Since the regime shift in 1999, both the areal extent and volume of hypoxia and anoxia have been elevated to levels never recorded before (Hansson et al. 2011).

## Summary

Fine-grained sediments of the study area act as NP and OP sinks. A comparison of the calculated inventories of OP and NP showed clear regional differences, with the highest values in the SE part of the Gotland Deep (610 μg m^2^ of NP and 47 μg m^2^ of OP) and the lowest values at the slope of the Gdansk Deep (24 μg m^2^ of NP and 16 μg m^2^ of OP). Such spatial distribution was probably, among other factors, the result of the uplift of the Baltic Sea floor.

Although in detail the alkylphenols showed individual differences in the vertical distribution, an increase in concentrations since the 1960s/1970s of the twentieth century occurred for most of the studied regions. This period coincides with the first signs of eutrophication in the Baltic.

The youngest deposits, from about the past 10 to 15 years, were usually characterized by sharp increasing trends in the OP and NP, which could be associated with the increase in the areal extent and volume of hypoxia and anoxia in the Baltic Sea. The occurrence of OP and NP in sediment layers deposited before the mass production of alkylphenols could be associated with their infiltration in sediment column.

The results of the present study suggested that concentrations of alkylphenols in sediments were influenced by various complex factors. We can expect that even a considerable decrease in the external supply of alkylphenols to the Baltic Sea will not cause quick improvement of environmental conditions because of eutrophication and oxygen deficits in Baltic, which stimulate accumulation of alkylphenols in sediment.

## Electronic supplementary material

Below is the link to the electronic supplementary material.

Fig. 1S Profiles of 210Pbexp in sediment of study area (XLSX 24 kb)

Fig. 2S Vertical changes of 137Cs activity with the age of the subsequent sediment layers in study area (XLSX 14 kb)

Fig 3S Relationship between NP and OP concentration in sediment (XLSX 15 kb)
